# Exploration of the safe water content and activity control points for medicinal and edible lotus seeds from mildew

**DOI:** 10.1186/s13568-020-01019-1

**Published:** 2020-05-12

**Authors:** Xiaofang Liao, Chaonan Sun, Fang Wei, Lidong Zhou, Weijun Kong

**Affiliations:** Institute of Medicinal Plant Development, Chinese Academy of Medical Sciences & Peking Union Medical College, Beijing, 100193 China

**Keywords:** Lotus seeds, Mildew, Aflatoxins, Water content and activity, Relative humidity, Safe storage

## Abstract

Affected by the inner properties and the external environmental conditions, medicinal and edible lotus seeds are susceptible to mildew with fungal infection under suitable temperature and humidity conditions, leading to the production and contamination of various mycotoxins, along with threats to its quality and safety. In this study, the changes of water content (C_w_) and water activity (A_w_) of lotus seeds stored at 25 °C and different relative humidity conditions, as well as the correlation between them and mildew of this edible and medicinal material were studied, aiming to explore the safe C_w_ and A_w_ control points for screening out the suitable storage conditions from mildew. Blank (without fungal conidia) and experimental (artificially added with *Aspergillus flavus* conidia) groups of lotus seeds were stored at 25 °C and relative humidity of 40%, 50%, 60% and 70% for about 30 days, respectively. The mildew was observed and the changes of C_w_, A_w_, together with the production of aflatoxins were measured. Results showed that no mildew was found and aflatoxins were not detected in lotus seeds when they were stored for 30 days at 25 °C and relative humidity of 40%, 50% and 60% with C_w_ < 12% and A_w_ < 0.6. While, when the relative humidity was up to 70%, the C_w_ and A_w_ values rose quickly, and the C_w_ exceeded the officially-permitted level (14%). Although no mildew was observed, AFB_1_ was still detected, increasing the potential risk of lotus seeds regarding aflatoxins. For warranting the quality with economic and safe storage, lotus seeds are suggested to be stored at 25 °C and relative humidity lower than 60% with 12% and 0.6 as the safe C_w_ and A_w_ control points, respectively, to prevent medicinal and edible products from mildew and the contamination of aflatoxins.

## Introduction

Foods including medicinal and edible products, feeds and medicinal plants are vulnerable to fungal conidia and mildew if improperly treated in the processes of growth, harvest, processing, storage and transportation (Giorni et al. 2018; Liu et al. 2015b; Zhang et al. 2019). Under suitable temperature (20–35 °C) and humidity (over 70% or water content more than 15%) and sufficient nutrient conditions, these fungal conidia can sprout mycelium, secrete enzymes, and then dissolve foods or medicinal plants and decompose effective ingredients of them, reducing their quality and efficacy (Liu et al. 2015b). At the same time, some toxigenic fungi can produce secondary metabolites-mycotoxins with serious toxicity (Abarca et al. 2019; Dawit et al. 2019; Giorni et al. 2018; Jelena et al. 2019; Lv et al. 2019; Wang et al. 2009) to threaten their quality and safety, along with the physical and mental health of the consumers.

Various mycotoxins have been found with residue in different kinds of matrices (AlFaris et al. 2019; Kuang et al. 2013; Li et al. 2016; Liu et al. 2012; Su and Pan 2018; Wei et al. 2019; Xie and Li 2016). Among them, lotus seed (*Nelumbo nucifera* Gaertn), as a valuable and commonly-used edible and medicinal matrix, not only has the properties of tonifying spleen, stopping diarrhea, stopping bandage, tonifying kidney, nourishing heart and tranquilizing mind, but also is a common food or food additive with wide nutritional and edible functions. However, affected by the nature of their components and the external environmental conditions, lotus seeds are susceptible to mildew with fungal infection under suitable temperature and humidity conditions, especially during the plum rain season, leading to the production and residue of various mycotoxins, such as aflatoxins, ochratoxins, etc. Aflatoxins, mainly containing aflatoxin B_1_ (AFB_1_), B_2_ (AFB_2_), G_1_ (AFG_1_), and G_2_ (AFG_2_) with strong hepatotoxicity and carcinogenic, teratogenic and mutagenic effects (Peckham et al. 1971), have been classified as Group 1A carcinogens by the International Agency for Research on Cancer (IARC) (World Health Organization [WHO] and International Agency for Research on Cancer [IARC] 1993). Liu et al. (2013) found that 95% (19/20) lotus seeds samples were contaminated with aflatoxins at levels ranging from 0.02 to 6888.4 µg/kg and AFB_1_ was the predominant aflatoxin. Another study (Liu et al. 2019) regarding 57 lotus seed samples showed that AFB_1_ had the highest incidence of 26.3% with residual level from 0.25 to 7.48 µg/kg. Similar results were also reported by Wei et al. (2019). Taking their prominent medicinal value and edible function, as well as the high occurrence of mildew and aflatoxins contamination into consideration, a maximum residue level (MRL) of 5 µg/kg for AFB_1_ and 10 µg/kg for the total amount of AFB_1_ + AFB_2_ + AFG_1_ + AFG_2_ in lotus seeds (Chinese Pharmacopoeia Commission 2015a) have been officially set. Therefore, it is of great significance and urgency to prevent lotus seed from mildew and aflatoxins production through controlling the crucial factors or conditions, such as water content (C_w_), water activity (A_w_) and environmental temperature and humidity, to ensure their quality and safety.

It is generally believed that in the processing, storage and transportation processes, foods, medicinal plants and other matrices should be dried sufficiently to control their water content for inhibiting the growth of mildew or pests. In addition, A_w_ (Rockland and Beuchat 1987) is also an important indicator that needs to be focused on and controlled by the dynamic measurement of water energy in foods, indicating the extent to which water can be used by microorganisms including toxigenic fungi. A_w_ is expressed by the ratio of vapor pressure of water in the matrix to saturated vapor pressure of pure water, which is equal to the relative humidity of air above the sample. In addition, A_w_ is directly related to the sensitivity of microorganisms in foods, and has a high correlation with the chemical and physical reactions of degradation on the shelf life of foods. And A_w_ can be introduced to predict the longest shelf life (John et al. 2019), as well as to determine the packaging conditions and storage time of foods, etc. In some important regulations and guidance documents, A_w_ has been the only factor that can be measured and quantified in the Hazard Analysis Critical Control Point (HACCP) system. In practice, foods are commonly dried to reduce the water content to prevent them from mildew. In the other hand, salt or sugar is added to decrease the A_w_ of matrices to inhibit the growth of toxigenic fungi. Therefore, in public view, effective control C_w_ and A_w_ could prevent foods from mildew and the production of mycotoxins to ensure their safety.

**Fig. 1:**
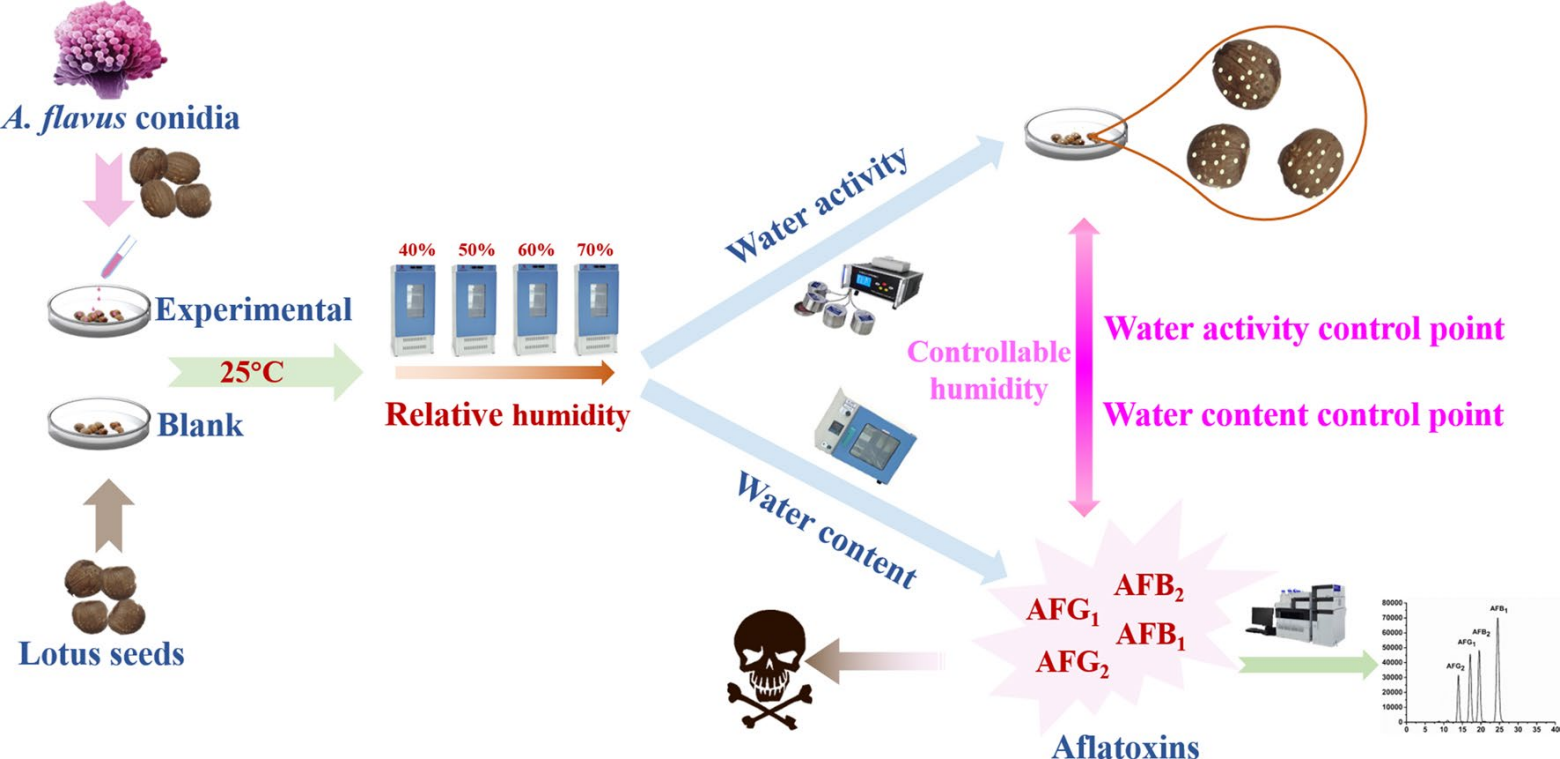
Schematic diagram on exploration of the correlation between mildew and water content and activity of lotus seeds

Therefore, through the investigation of the C_w_ and A_w_ changes of lotus seeds under different storage conditions according to the experimental design in Fig. 1, we aimed to study the correlation of C_w_ and A_w_ and mildew for exploring the optimum water content and water activity control points for the safe storage. These findings will provide powerful supports and valuable references for screening for the reasonable, reliable and effective storage conditions to prevent medicinal and edible foods and other products from mildew and mycotoxins contamination for ensuring their quality and safety.

## Materials and methods

### Chemicals and reagents

The mixed aflatoxins standard solution containing 2.0 mg/mL of AFB_1_ and AFG_1_, and 0.5 mg/mL of AFB_2_ and AFG_2_ was purchased from Pribolab Pte. Ltd. (Singapore). The ToxinFast^®^-Aflatoxins Test immunoaffinity columns were bought from Huaan Magnech Bio-Tech Co., Ltd (Beijing, China). The aflatoxigenic * Aspergillus flavus (A. flavus)* lyophilized powder (CGMCC 3.4410) were got from the China General Microbiological Culture Collection Center (Beijing, China) and cultured to 1 × 10^7^ conidia/mL suspension for use.

HPLC-grade acetonitrile and methanol were obtained from Thermo Fisher Scientific Inc (Fair Lawn, NJ, USA). NaCl, KCl, Na_2_HPO_4_.12H_2_O, NaH_2_PO_4_, KH_2_PO_4_, and tween-20 were of analytical grade and purchased from Sinopharm Chemical Reagent Co., Ltd (Shanghai, China). Purified Wahaha water from Wahaha Group Co., Ltd (Hangzhou, China) was used for all the experiments.

### Sample collection and treatment

Lotus seed (*Nelumbo nucifera* Gaertn.) samples, produced in Hubei province, (batch number 17050040, China) were purchased from Beijing Tong Ren Tang Co., LTD. All the samples were divided into the blank and experimental groups. For the blank groups: 20 g lotus seeds were added into 16 petri dishes, respectively, followed by irradiation for 5 h under an ultraviolet lamp. For the experimental groups: 20 g lotus seeds were added into another 16 petri dishes, respectively. After irradiation for 5 h, 1 mL of *A. flavus* conidia solution was artificially added onto the surface of lotus seeds and were air-dried. Then, all the petri dishes containing the raw and treated samples were stored in an incubator at 25 °C and relative humidity conditions of 40%, 50%, 60% and 70%, respectively. On the 0, 10th, 20th and 30th day, all the samples were observed by naked eyes regarding mildew. In addition, the samples were treated for the measurement of A_w_ and C_w_, along with the determination of potential aflatoxins by the optimized high performance liquid chromatography coupled with photochemical derivatization and fluorescence detection (HPLC-PCD-FLD) method on the 0, 3rd, 6th, 9th, 12th, 22nd and 32nd day.

### Water content determination

The weight loss method recommended in the Chinese Pharmacopoeia (Chinese Pharmacopoeia Commission 2015b) was used. 2–5 g raw materials of lotus seed were accurately weighed and tiled in a flat weighing bottle with the thickness less than 5 mm. After the samples were dried at 100–105 °C for 5 h to constant weight, they were transferred to a dryer for cooling for 30 min, followed by another drying at 100–105 °C for 1 h and cooling until the difference between the two successive weighing was not more than 5 mg. The water content (C_w_, %) of sample was accurately calculated based on the lost weight.

### Water activity measurement

Five gram lotus seeds out of the above samples stored at 25 °C and different relative humidity (40%, 50%, 60% and 70%) conditions were taken out on the 0, 3rd, 6th, 9th, 12th, 22nd and 32nd day for the measurement of water activity (A_w_) by using a HD-6 (Wuxi Huake Instrument, Wuxi, China) intelligent moisture activity measuring instrument. 15 min later, data of A_w_ were recorded.

### Aflatoxins determination

#### Apparatus and high-performance liquid chromatography-fluorescence detection conditions

Chromatographic separation of four aflatoxins was performed on a MG-III-C18 column (4.6 mm × 150 mm, 5 μm) through a Shimadzu LC-20AT HPLC system (Shimadzu, Kyoto, Japan) consisting of two LC-20 AT pumps, an SIL-20A autosampler, a CTO-20A column oven, a CMB-20A controller, the post-column photochemical derivatization (PCD) reactor and an RF-20AXL fluorescence detector (FLD). Methanol–acetonitrile (40:18, *v/v*) and water was selected as the mobile phase with isocratic elution at a flow rate of 1.2 mL/min. The column temperature was set at 30 °C. The injection volume was 20 μL. The AURA INDUSTRIES PCD reactor (New York, NY, United States) was consisted of a mercury lamp (λ = 254 nm) and a knitted reactor coil of 0.74 mL (15 m × 0.25 mm). The eluate was monitored by using a fluorescence detector at an excitation wavelength of 360 nm and an emission wavelength of 450 nm.

#### Sample preparation for four aflatoxins determination

Firstly, Phosphate buffer saline (PBS) solution was prepared by dissolving 0.2 g KCl, 8.0 g NaCl, 0.2 g KH_2_PO_4_, 2.9 g Na_2_HPO_4_·12H_2_O in 1000 mL of purified water. 2% PBT (phosphate buffer with addition of Tween-20) solution was obtained by dispersing 0.2599 g NaH_2_PO_4_, 6.4204 g Na_2_HPO_4_.12H_2_O and 40 mL of tween-20 in 1960 mL of purified water.

Then, 5 g homogenized lotus seed sample powders that have been stored at 25 °C and relative humidity of 40%, 50%, 60% and 70% for 30 days were accurately weighed and placed in a 50-mL centrifuge tube with the addition of 1 g NaCl and 25 mL of methanol–water (80:20, *v/v*) solution. After blending by vortex and ultrasonication for 30 min, the mixture was followed by centrifugation for 5 min at 10,000 r/min and 5 mL of the supernatant was collected and transferred into a 50-mL EP tube containing 40 mL of 1% PBT solution. The solution was mixed with pH being adjusted to 6–8, followed by filtration through a 0.45-µm syringe nylon filter. 40 mL of the filtrate was precisely measured and elutriated through an immunoaffinity column at a flow rate of 1–2 drop(s) per second, then 10 mL of PBS and water were taken, respectively, to wash the column successively, and finally 800 µL of methanol was added to elute the target aflatoxins. 1 mL of the eluent was collected and filtered through a 0.22-µm syringe nylon filter for HPLC-PCD-FLD analysis.

#### Establishment of standard curves of four aflatoxins

The mixed standard solution of aflatoxins was diluted to 9 different concentrations of working solutions, and 20 µL of the dilution was injected successively from low concentration to high concentration into the HPLC-PCD-FLD system. The peak area of each aflatoxin was recorded and the standard curve was established by linear regression of the peak area (*y*) *versus* the injection concentration (*x*). Then, the contents of aflatoxins in the tested samples were quantified according to the standard curves. All the working solutions were freshly prepared just before being injected into the HPLC analytical system.

#### Statistical analysis

All data regarding C_w_ and A_w_ were expressed as mean ± standard deviation (SD) and analyzed by using the Origin 2016 software.

## Results

### Changes of water content of lotus seeds during storage

**Fig. 2:**
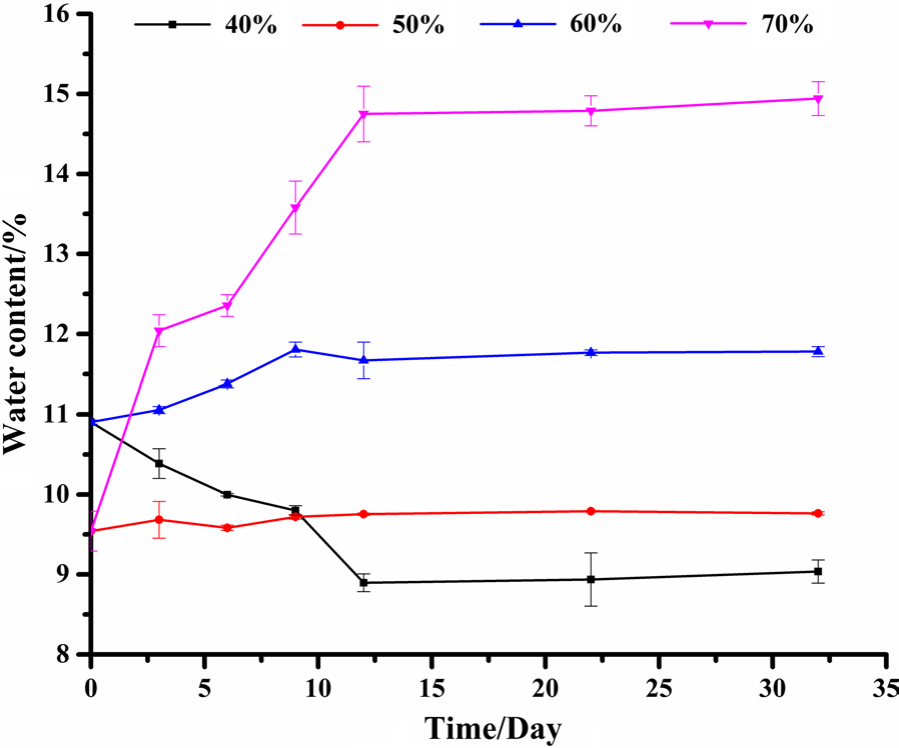
Water content of lotus seeds stored at 25 °C and different relative humidity conditions

The C_w_ of lotus seeds stored at 25 °C and different relative humidity conditions of 40%, 50%, 60% and 70% within 32 days was measured and shown in Fig. 2. It could be found that the C_w_ values exhibited a fluctuation under different relative humidity conditions within the first 10 days, and then were relatively stable during the next 22 days, which was increased successively from low humidity to high humidity. The higher the relative humidity was, the bigger the C_w_ value was. The samples stored at relative humidity 70% exhibited the highest C_w_ values. After storage for 12 days at 25 °C and relative humidity 70%, the C_w_ value exceeded the prescribed limit (14%) for lotus seeds in the Chinese Pharmacopoeia (Chinese Pharmacopoeia Commission 2015b). In addition, it could be found that when the relative humidity was lower than 70%, the C_w_ values were all not more than 12%, which might be suggested as the suitable water content control point for safe storage of lotus seed in practice. These findings indicated that the relative humidity conditions for the storage environment of lotus seeds should be effectively controlled to lower the water content (not more than 12%), and further to prevent this medicinal and edible food from mildew.

### Changes of water activity of lotus seed during storage

**Fig. 3:**
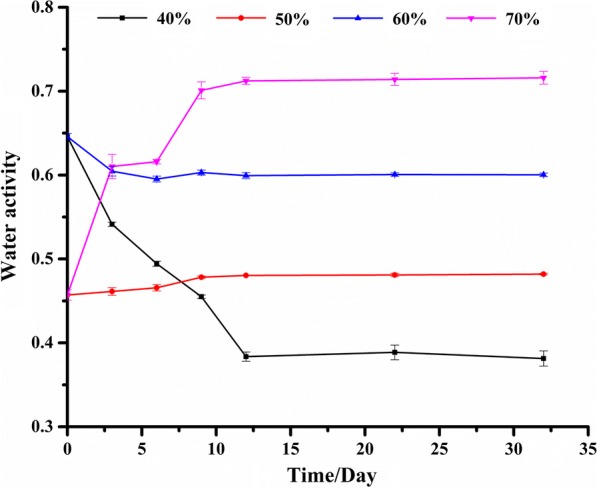
Water activity of lotus seeds stored at 25 °C and different relative humidity conditions (40%, 50%, 60% and 70%)

The water activity (A_w_) data of lotus seeds stored at 25 °C and different relative humidity conditions of 40%, 50%, 60% and 70% within 32 days were determined and listed in Fig. 3. It could be observed that the changes of A_w_ values expressed a similar trend with C_w_, which presented some fluctuation under different relative humidity conditions within the first 10 days, and then were stable during the next 22 days. After storage for 10 days at 25 °C, the A_w_ values were increased with increasing the relative humidity from 40% to 70%, which reached the highest values at relative humidity 70%. After storage for 12 days at 25 °C and relative humidity 70%, the A_w_ values were bigger than 0.7, which exceeded the relative humidity value of 70%. When the relative humidity condition was lower than 70%, the A_w_ values were all smaller than 0.6, which might be considered as the suitable water activity control point for safe storage of lotus seed in practice. These data illustrate that the increase of relative humidity of storage condition will lead to the enhancement of the water activity of lotus seeds, which may raise the incidence of mildew of this edible and medicinal food. While, from then on, the maximum limit standard, as well as some regulations on A_w_ for lotus seed and other matrices are lacking. To prevent lotus seeds from mildew and mycotoxins contamination, it is suggested that the relative humidity conditions for the storage environment of lotus seed should be effectively controlled to lower the A_w_ in an acceptable range (not more than 0.6).

### Mildew of lotus seed during storage

**Fig. 4:**
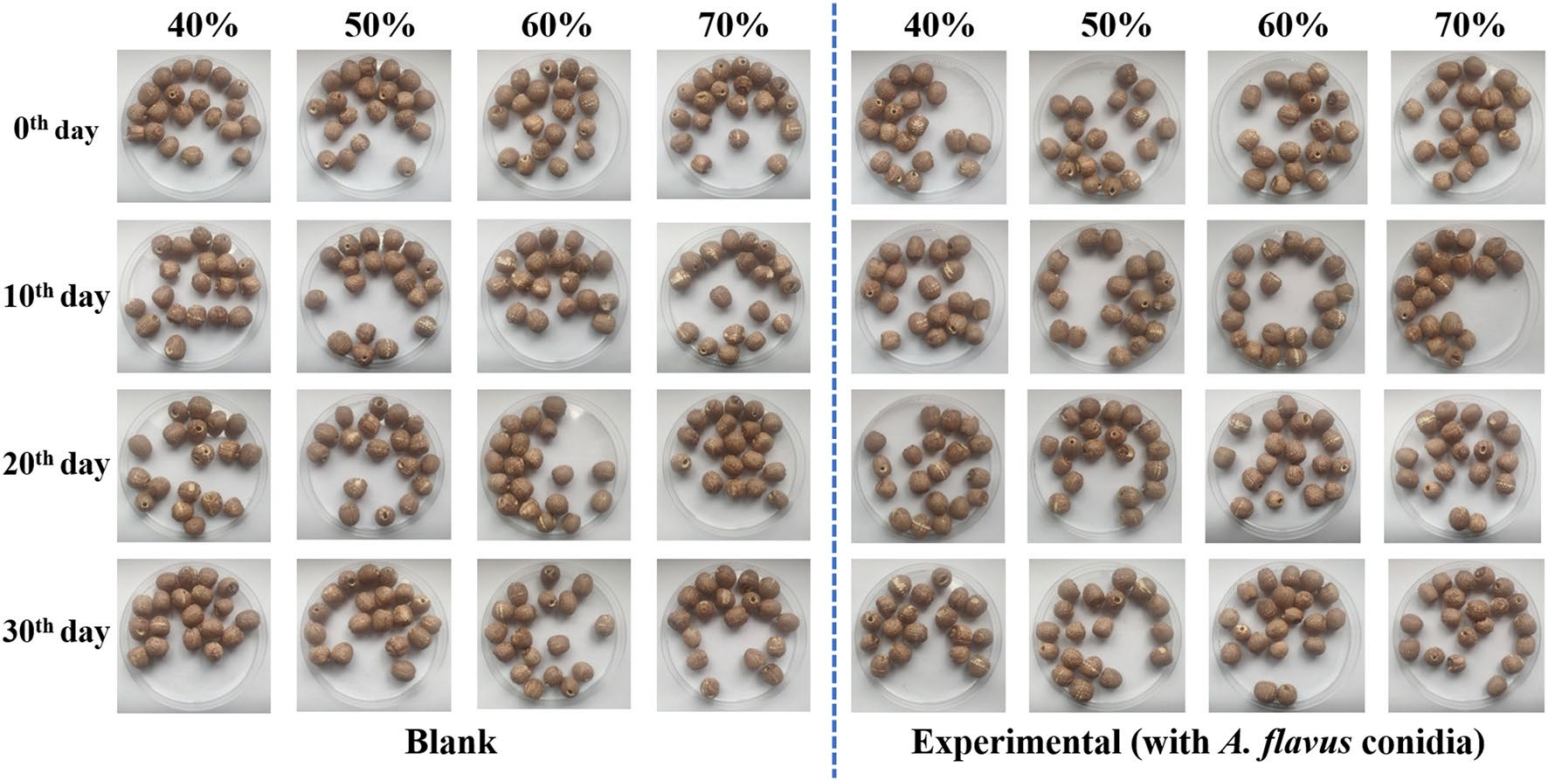
Mildew changes of lotus seed stored at 25 °C and different relative humidity conditions (40%, 50%, 60% and 70%)

The above results have shown that the C_w_ and A_w_ values were both increased with increasing the storage relative humidity from 40% to 70% at 25 °C. Here, the mildew on the surface of blank lotus seeds (without fungal conidia) and experimental samples (inoculated with *A. flavus* conidia that could produce aflatoxins) stored at 25 °C and relative humidity 40%, 50%, 60% and 70% for 30 days was observed at an interval of 10 days. It could be seen in Fig. 4 that no visible mildew was found on the surface of all samples. Nevertheless, aflatoxins might be produced, which would be detected in the next part.

### Determination of aflatoxins

#### Standard curve of four aflatoxins

**Fig. 5:**
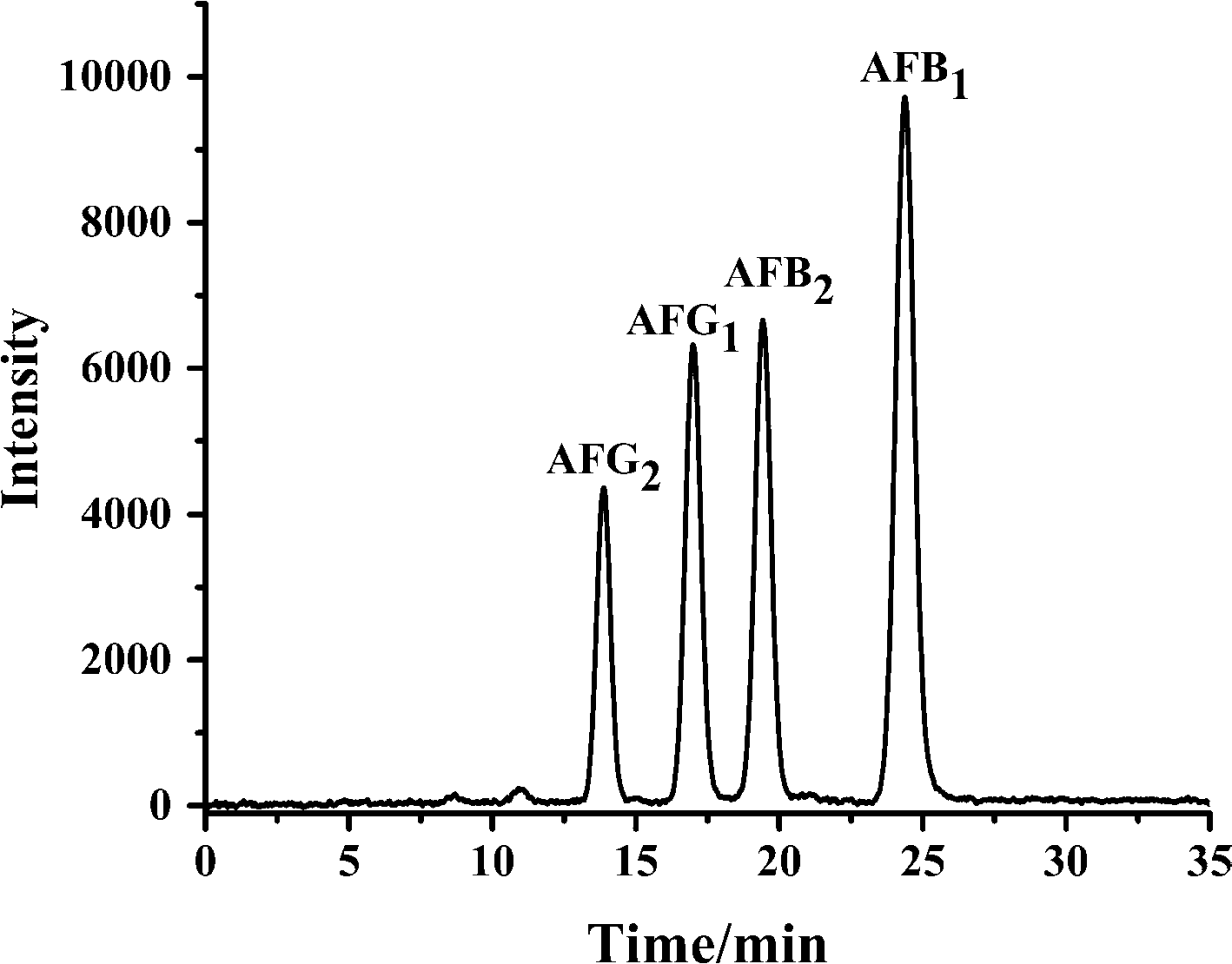
HPLC-PCD-FLD chromatogram of four aflatoxins

Under the optimized chromatographic conditions, the HPLC-PCD-FLD chromatogram of four aflatoxins was recorded and shown in Fig. 5. Then, the peak area values (*y*_*B1*_, *y*_*B2*_, *y*_*G1*_, *y*_*G2*_) of four aflatoxins at different concentrations (*x*) were determined and the linear regression equations with their correlation coefficient (*R*^*2*^), as well as the limit of detection (LOD) and quantitation (LOQ) were obtained and listed in Table 1. The results indicated that the developed HPLC-PCD-FLD method was sensitive and reliable for accurate quantitation of four aflatoxins in real samples within wide concentration ranges.

**Table 1:** Regression equation, LOD and LOQ of four aflatoxins

AFs	Regression equation	*R*^*2*^	Linear range (ng/mL)	LOD (μg/kg)	LOQ (μg/kg)
AFB_1_	*y*_*B1*_= 33,420 *x* + 49,526	0.9999	0.78125–200.0	0.30	0.88
AFB_2_	*y*_*B2*_ = 76,477 *x* + 30,833	0.9999	0.39063–50.0	0.15	0.44
AFG_1_	*y*_*G1*_ = 17,988 *x* + 26,881	1.0000	0.78125–200.0	0.30	0.88
AFG_2_	*y*_*G2*_ = 43,679 *x* + 19,772	0.9998	0.39063–50.0	0.15	0.44

#### Changes of aflatoxins contents in lotus seeds during storage

Although no visible mildew was observed on the surface of lotus seeds by naked eyes within 30 days, it was not definite if toxic aflatoxins were produced in the samples. Herein, the lotus samples stored at 25 °C and relative humidity of 40%, 50%, 60% and 70% for 0, 10, 20 and 30 days were collected and prepared for aflatoxins determination using the developed HPLC-PCD-FLD method. The results in Table 2 showed that no aflatoxins were detected in lotus seed samples of both the blank and experimental groups when the relative humidity conditions were set at 40%, 50% and 60%. While, when the relative humidity was up to 70%, AFB_1_ was detected in both the blank and experimental lotus seed samples after they were stored at 25 °C for 30 days, and the concentration in the samples of experimental group reached 6.6 μg/kg, which has exceeded the officially-recommended MRL (5.0 μg/kg).

**Table 2:** AFs detected in lotus seeds during storage at 25 °C and different relative humidity conditions

Average levels of AFs at different storage time points (μg/kg, *n *= 3)								
Relative humidity	Day 0		Day 10		Day 20		Day 30	
	Blank group	Experimental group	Blank group	Experimental group	Blank group	Experimental group	Blank group	Experimental group
40%	**–**	**–**	**–**	**–**	**–**	**–**	**–**	**–**
50%	**–**	**–**	**–**	**–**	**–**	**–**	**–**	**–**
60%	**–**	**–**	**–**	**–**	**–**	**–**	**–**	**–**
70%	**–**	**–**	**–**	**–**	**–**	**–**	< LOQ (AFB_1_)	6.6^a^ (AFB_1_)

^a^RSD < 0.2%

These results indicated that lotus seed was a susceptible matrix to mildew and aflatoxins contamination. Although no visible mildew was observed, AFB_1_ could still be detected in lotus seeds stored for long time (about 30 days) at 25 °C and high relative humidity (around 70%), especially in the samples without incubation with toxigenic fungi, which would increase the risk incidence of potential safety threatens of lotus seeds, and should be given more attention. Therefore, in practice, the relative humidity of storage environment for lotus seeds should be effectively controlled in less than 70% with the optimum water content and activity control points at 12% and 0.6, respectively, for safe storage to ensure the quality and safety of this edible and medicinal food related products, as well as the health of consumers.

## Discussion

Fungal spoilage and mycotoxin contamination are a major problem for many medicinal and edible foods and traditional Chinese medicines (TCMs). If storage conditions are poorly managed, some fungi species can infect these matrices, leading to mycotoxin contamination. Environmental temperature and humidity conditions are the two key factors for the growth of fungi, which should be in effective control to prevent foods, feeds and medicinal plants from mildew. In common, most fungi will grow and multiply quickly at 20–35 °C and relative humidity more than 70% (Liu et al. 2015a, b). High environmental humidity will lead to the increase of C_w_ and A_w_ of the matrices for fungal growth, further may result in the occurrence of mildew and mycotoxins contamination (Abarca et al. 2019; Nian et al. 2018; Qiu et al. 2015; Wang et al. 2008; Xu et al. 2017; Yang et al. 2017; Zheng et al. 2016). Liu et al. (2015a) found that *Areca catechu* was not susceptible to mildew infection or mycotoxins production in the environment with humidity below 90% and temperature under 25 °C. The best storage conditions for *Radix Astragali* and *Alpinia oxyphylla* to avoid *Aspergillus flavus* contamination were temperature and humidity below 25 °C and 85%, respectively (Hu et al. 2015; Zhao et al. 2017).

Lotus seeds contain approximately 500 g/kg (dry basis) starch (Zhang et al. 2014; Wang et al. 2018; Lei et al. 2020), as well as up to 19.85% protein (Zeng et al. 2013), which provide a large amount of nutrients for the growth and reproduction of fungi like *Aspergillus flavus*, under suitable experimental conditions. Therefore, it is of great significance to study the critical conditions for lotus seeds to mildew and infect aflatoxins. In this study, the blank (untreated samples) and experimental (artificially-contaminated samples with aflatoxigenic *A. flavus* conidia) samples of lotus seeds were stored at 25 °C and different relative humidity conditions to measure the C_w_ and A_w_ changes. Our results have shown that both the C_w_ and A_w_ values tended to be stable after storage for 10 days, and no obvious mildew was observed on the surface of all tested samples for 30 days. When the relative humidity was up to 70%, the C_w_ and A_w_ values were increased quickly, and the C_w_ value exceeded the official-permitted level (14%), which was in agreement with the reports (Liu et al. 2015b). Within 30 days, although mildew was not observed, AFB_1_ could still be detected, which would increase the potential risk of lotus seeds regarding aflatoxins.

In the practical medicinal and edible foods and TCMs storage process, the experimental temperature and humidity, as well as the C_w_ and A_w_ conditions were seldom paid special attention and failed to control. Changes of weather and environmental conditions will lead to large humidity fluctuations. When the environmental humidity is above 70% for a long time, the water content and activity of foods will be high, which will easily result in the mildew of TCMs, along with mycotoxins contamination. Our results have exhibited that when the relative humidity was no more than 60%, the C_w_ and A_w_ values approximately equal to about 12% and 0.6, respectively, which might be recommended as the optimum water content and water activity control points for safe storage of lotus seeds in practice at 25 °C with the environmental relative humidity less than 60%, to ensure the quality, safety and effectiveness of medicinal and edible products, as well as the health of the consumers.

## Data Availability

All data in this manuscript were deposited in publicly available repositories in the Institute of Medicinal Plant Development, Chinese Academy of Medical Sciences & Peking Union Medical College, Beijing, China.
